# Suicide among older adults in Ireland: a national case series of coronial data, 2015–2020

**DOI:** 10.1136/bmjment-2026-302665

**Published:** 2026-07-14

**Authors:** M Isabela Troya, Paul Corcoran, Anne M Doherty, Katerina Kavalidou, Caoimhe Lonergan, Faraz Mughal, Ella Arensman, Michael J Norton, on behalf of the Lived Experience Advisory Group, Jim Blighe

**Affiliations:** 1 School of Public Health, University College Cork, Cork, Ireland; 2 National Suicide Research Foundation, Cork, County Cork, Ireland; 3 Psychiatry, University College Dublin School of Medicine, Dublin, Ireland; 4 Department of Psychiatry, University College Cork, Cork, Ireland; 5 Nuffield Department of Primary Care Health Sciences, University of Oxford, Oxford, UK; 6 Adult Continuing Education, University College Cork, Cork, Ireland; 7 Recovery and Engagement Programme Lead, Office of Mental Health Engagement and Recovery, Health Service Executive, Dublin, Ireland

**Keywords:** Mental Health, Community Mental Health Services, Mental Health Services

## Abstract

**Background:**

Older adults have the highest suicide rates globally, yet contemporary national evidence on suicide in later-life remains limited in Ireland.

**Objective:**

To examine the rates, sociodemographic and clinical factors of older adults who die by suicide in Ireland.

**Methods:**

Retrospective analysis of coronial files of older adults aged ≥60 years who died by suicide in Ireland during 2015–2020, with adults aged 18–59 years as a comparison group. Crude suicide rates per 100 000 were calculated. Differences in sociodemographic and clinical variables were tested using χ^2^ or Fisher’s exact tests. ORs with 95% CIs compared suicide methods between age groups. Poisson regression models estimated changes in suicide rates. A Lived Experience group contributed to interpretation.

**Findings:**

Between 2015 and 2020, 654 older adults died by suicide. The average suicide rate for older adults was 12.0 per 100 000 compared with 17.5 per 100 000 among adults aged 18–59. However, marked within-group variation existed, with older single males having the highest suicide rate overall (38.1 per 100 000). Among older adults, males aged 60–69 years had the highest rate (21.8 per 100 000). Suicide risk increased during summer, especially among older adults. Rates declined in 2020 in older males. Among individuals who died by suicide, older adults were more likely to be married, live alone and in agricultural and managerial socioeconomic groups compared with adults aged 18–59. Among suicide deaths, older adults were more likely to have physical health conditions, recent acute pain, loneliness and seen their general practitioner (GP). Within the suicide cohort, financial concerns and interpersonal difficulties were more commonly experienced by younger adults. Method profiles differed by age, with drowning and firearm-related suicides higher among older adults.

**Conclusions:**

Older adults who died by suicide in Ireland had distinct sociodemographic and clinical profiles compared with younger adults. While mental health symptoms were common across age groups, later-life suicide was more frequently characterised by physical health conditions, loneliness and recent healthcare contact.

**Clinical implications:**

Findings highlight the need for sex-specific and age-specific suicide prevention strategies that address systemic factors, including improved GP training and strengthened community-based services. Increased contact with healthcare services highlights opportunities for intervention.

**WHAT IS ALREADY KNOWN ON THIS TOPIC**

- Globally, suicide rates are highest among older adults, in particular older males; however considerable regional variation exists.

**WHAT THIS STUDY ADDS**

- Using 6 years of national coronial data (2015–2020), findings highlight the distinct clinical and sociodemographic profile of older adults who die by suicide in Ireland when compared with adults aged 18–59 years. Older adults aged 60–69 years, especially single males and those transitioning to retirement, represent a key group for targeted prevention. Reductions in male suicide rates were observed in 2020 across both age groups. Seasonal variation in suicide rates was observed, with an increased risk during summer months in both age groups; this increase was more pronounced among older adults, particularly males.

**HOW THIS STUDY MIGHT AFFECT RESEARCH, PRACTICE OR POLICY**

- Findings support the need for sex-specific and age-specific suicide prevention strategies and suggest the importance of integrated approaches across primary care, specialist services and accessible community-based supports for older adults including social prescribing initiatives and culturally appropriate programmes for isolated older adults.
- Improved routine screening for suicidality and psychosocial distress in older populations is needed considering older adults were more likely to have recent contact with health services, including in general practice.

## Introduction

 Suicide, defined by the WHO as the act of deliberately killing oneself,^1^ is a major public health concern. Globally, suicide rates increase with age, with the highest rates observed among older people.^2 3^ In 2021, global suicide rates were estimated at 9.0 per 100 000 for all age groups, with older adults aged 70 and older having the highest mortality rates (males: 37.9 and females: 15.6 per 100 000).^2 3^ This study defines older adults as those aged 60 years and older, consistent with WHO definitions.^4^ However, internationally suicide data are typically reported for broader age categories (eg, 70 years and older), and as such these are used to contextualise the elevated risk in later-life. Social connectedness, resiliency and purpose in life are protective factors for suicidality in later life;^5^ however, older adults have high suicide rates which have been found to be associated with a range of psychosocial and health-related factors including social isolation, physical illness, physical impairment, bereavement, psychiatric conditions and financial stressors.^6 7^ Despite older adults having the highest suicide rates worldwide, there is considerable regional variation.

In Ireland, the mental health of older people is considered a national priority^8^ as is suicide prevention.^9^ National research has examined hospital self-harm trends among older adults^10^ and established suicide risk following hospital-presenting self-harm, with older age identified as a key risk factor.^11^ However, the most recent Irish study examining the characteristics of older adults who died by suicide analysed data up to 2006,^12^ and thus an update is timely and can help inform future Irish national policy and practice. Since then, Ireland has experienced significant demographic ageing alongside changing global suicide patterns. While hospital-based studies provide important insights into non-fatal self-harm, updated national mortality data are required to capture suicide deaths among adults aged 60 years and older and to inform contemporary policy and practice. This study contributes to the United Nations Sustainable Development Goal 3, particularly Target 3.4, which includes reducing suicide mortality as a key indicator of progress in mental health, by strengthening evidence for suicide prevention in a high-risk but often underrepresented population. By providing contemporary national coronial data, this study establishes a necessary descriptive baseline to inform clinical practice, service planning and future analytical research on pathways to suicide in later-life.

This study aims to examine the sociodemographic and clinical factors and rates of older adults who die by suicide in Ireland using coronial data from the Irish Probable Suicide Deaths Study (IPSDS), with comparisons made to adults aged 18–59 years. Based on evidence from previous Irish research^12^ we hypothesised there would be some sociodemographic (relationship status, living arrangements and socioeconomic group) and clinical (contact with health services, history of mental health condition) differences among the two age groups, and that suicide rates among adults aged 18–59 years would be higher compared with older adults, consistent with prior national findings.^11^

## Methods

### Study design and population

We conducted a case series study using retrospective national data of deaths by suicide in Ireland by older adults aged 60 years and older during 2015–2020 using coronial data from the IPSDS. A comparison group of adults aged 18–59 was included to contextualise suicide rates and characteristics observed among older adults and to identify age-specific differences relevant to prevention and service planning. We used data from 2015, the first year available in the IPSDS, to 2020, the most recent year at the time of this study’s submission. Report of this study is in line with STROBE (Strengthening the Reporting of Observational Studies in Epidemiology) guidelines, based on a published protocol that was followed throughout.^13^

### Data source

The IPSDS is a 6-year database that includes coroner and research-determined suicides, nationally, based on information from closed coronial files (2015–2020). The IPSDS includes sociodemographic, clinical and adverse life events that precede a suicide death, based on all relevant information that coroners collect during a death investigation. This national database was commissioned in response to obtaining further information and detail to suicides, compared with the basic descriptive suicide mortality data which can be accessed in the Central Statistics Office (CSO). The IPSDS follows the standardised data collection methodology of the National Drug-Related Deaths Index. Further details on the IPSDS database can be found in a study by Cox *et al.*^14^

### Outcome

The outcome is death by suicide. The IPSDS defines suicide as ‘all deaths with a coronial suicide verdict (which, by definition, are considered to be suicide ‘beyond reasonable doubt’); and deaths that are more likely than not, based on the weight of evidence, to have been a suicide (research determined suicide/on the balance of probabilities)’.^14^ All suicides and probable suicides included in the IPSDS will be called suicides hereafter.

### Measures

The IPSDS includes sociodemographic and clinical variables. The variables included in this study were restricted to those available within the IPSDS and were not selected based on a predefined conceptual framework; rather, they reflect the information recorded during coronial investigations and subsequently entered into the database. The IPSDS captures the following variables where available:

- Sociodemographic: sex, age, relationship status, living arrangements and socioeconomic group.
- Clinical: history of mental health condition (whether formally diagnosed or not), prescribed mental health medication, alcohol and drug use history, prior self-harm, contact with health services and adverse life events.
- Death detail: Coroner’s verdict, method of death, place of incident, suicide note left.

Age was recorded in completed years at the time of death and sex was recorded as noted in the coronial file. Other sociodemographic and clinical variables were derived from information documented during coronial investigations. These measures are obtained by a combination of sources by coroners from police forms, postmortem reports from pathologists, toxicology reports, medical reports, depositions from witnesses and/or family.^14 15^

Adverse life events reflect circumstances proximate to death documented in coronial records (eg, physical illness, recent acute pain, financial or interpersonal stressors) and are dependent on the availability and quality of information gathered during the coronial investigation.

### Statistical analysis

Descriptive statistics summarise all suicide deaths recorded in the IPSDS from 2015 to 2020. Frequencies and percentages were calculated for sociodemographic, clinical and death-related variables, stratified by age group (older adults aged ≥60 years vs adults aged 18–59 years). Differences in categorical variables between age groups were tested using the χ^2^ or Fisher’s exact test where expected cell counts were small (ie, <5). All analyses were conducted as bivariate comparisons, with variables examined independently. A p value of <0.05 was considered statistically significant. ORs with 95% CIs were calculated to compare method of suicide between the age groups, using individuals aged 18–59 years as the reference category.

To contextualise suicide among older adults in Ireland, crude suicide incidence rates per 100 000 population were calculated for the study period overall and stratified by age group (18–59 years and ≥60 years) and sex. For older adults, 10-year age groups were further calculated (60–69 years, 70–79 years, 80 years and older). Annual suicide rates were estimated using IPSDS suicide counts as numerators and national mid-year population estimates from the CSO as denominators. CSO census population data were used for 2016, and mid-year population estimates were used for 2015, 2017–2020. Exact Poisson 95% CIs were calculated for each rate.

Poisson regression models were used to estimate changes in suicide rates from 2015 to 2020 by sex and age group (10-year age group for older adults). The suitability of Poisson regression models was assessed using Pearson and deviance goodness-of-fit statistics, with non-significant results (p>0.05) indicating no evidence of model misfit or overdispersion. Incidence rate ratios (IRRs) alongside 95% CIs are reported, with 2015 used as the reference year.

Suicide rates by marital status ((1) married, cohabiting or civil partnership, (2) single, (3) separated or divorced, (4) widowed) were also calculated per 100 000 population by age group (18–59 years and ≥60 years) and sex across the study period. Population marital status data was available only for the census year, 2016, so we used 2016 CSO marital status data for all years. Poisson regression models examined differences in suicide rates by marital status and age group. IRRs alongside 95% CIs are reported, with married individuals used as the reference category.

As a post-hoc exploratory analysis, seasonal patterns in suicide deaths were examined using Poisson regression, with suicide counts as the outcome. Seasons were defined as winter (December–February; reference), spring (March–May), summer (June–August) and autumn (September–November), and analyses were stratified by sex and age group (18–59 years and ≥60 years). Models adjusted for differences in month length, and model fit was assessed using goodness-of-fit statistics.

Analyses were conducted using Stata V.19 and IBM SPSS V.29.

### Missing data

Given the nature of coronial data, some variables contained missing or unknown values. No imputation was performed. Analyses were conducted using available data for each variable. The extent of missing and unknown data is indicated in table footnotes.

### Patient and public involvement statement

A Lived Experience Advisory Group of seven individuals with experience of suicidality in older adults informed the research throughout. Five members reviewed the manuscript, providing feedback on language, sensitivity and interpretation of findings, which are presented with awareness of the experiences of older adults and bereaved families.

## Results

Between 2015 and 2020, 654 adults aged at least 60 years and a total of 2868 adults aged 18–59 years died by suicide in Ireland. The mean age for older adults was 68.1 (SD: 6.7, range: 60–97), while the mean age for adults aged 18–59 was 40.2 years (SD: 11.1).

### Suicide rates

**Figure 1:**
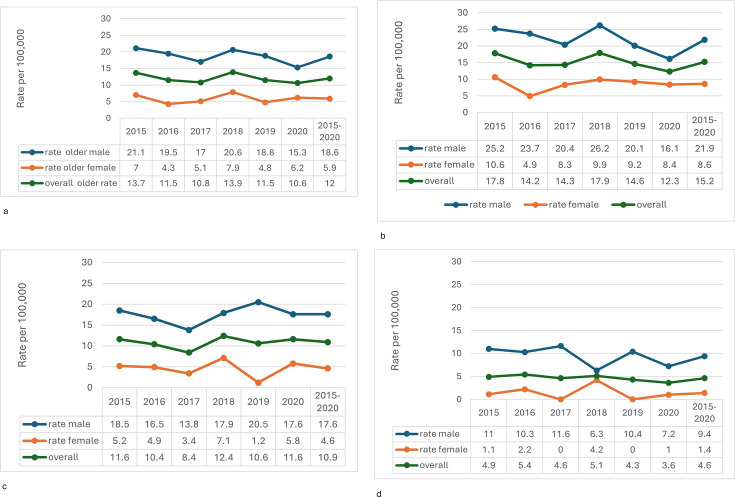
(a) Suicide rates among older adults 2015–2020, 60 years and older. (b) Suicide rate among older adults 2015–2020, 60–69 years. (c) Suicide rate among older adults 2015–2020, 70–79 years. (d) Suicide rate among older adults 2015–2020, 80 years and older.

From 2015 to 2020, the average suicide rate for older adults was 12.0 per 100 000 (see figure 1). Suicide rates were consistently higher among males than females in later-life: older males had a rate of 18.6 per 100 000, compared with 5.9 per 100 000 among older females during the 6-year study period. When stratified by age group, the highest suicide rates were observed among males aged 60–69 year-old (21.8 per 100 000), followed by males aged 70–79 years, while females aged 80 and older had the lowest suicide rate during the 6-year study period (1.4 per 100 000).

In comparison, adults aged 18–59 years had a higher overall suicide rate (17.5 per 100 000) over the 6-year study period, with rates remaining higher among males (26.5 per 100 000) than females (8.6 per 100 000) (see online supplemental file 1).

### Trends over the study period

Temporal trends in suicide rates among older adults were examined by age groups (60–69 years, 70–79 years and 80 years and older) and sex, using 2015 as the reference year (see online supplemental file 2). Overall, suicide rates among older adults were relatively stable over time, although some variation was observed by age and sex. Among older males aged over 60 years, suicide rates were significantly lower in 2020 compared with 2015 (IRR: 0.73, 95% CI 0.53 to 0.99, p=0.04).

Within the 60–69 age group, significant reductions were observed in 2016 among females (IRR: 0.46, 95% CI 0.22 to 0.94, p=0.03), and in 2020 among males (IRR: 0.64, 95% CI 0.42 to 0.96, p=0.03), as well as for the combined group overall (IRR: 0.69, 95% CI 0.49 to 0.96, p=0.03). In contrast, for older adults aged 70–79 years and those aged 80 years and older, IRRs varied across years but did not reach statistical significance, reflecting small numbers, particularly among females aged 80 years and older.

For comparison, patterns among adults aged 18–59 years were generally stable across the study period, with a single significant reduction observed in males 2020 (IRR 0.81, 95% CI 0.70 to 0.94, p<0.05).

### Seasonal variation

Among older adults (≥60 years), suicide rates were higher during summer (IRR 1.38, 95% CI 1.13 to 1.68, p=0.01) compared with winter. Seasonal effects were more pronounced among older males compared with older females (see online supplemental file 3).

Among adults aged 18–59 years, a comparable pattern was observed. Relative to winter, suicide rates were higher during summer (IRR 1.11, 95% CI 1.01 to 1.23, p=0.03). As in older adults, seasonal variation was more evident among males than females (see online supplemental file 3). Comparison of IRRs across age groups indicates that the summer increase in suicide rates was more pronounced among older adults than among adults aged 18–59 years.

### Rates by marital status

We examined suicide rates by marital status, age group and sex (see online supplemental file 4). The highest suicide rate observed among both age groups was in older single males: 38.1 per 100 000 (compared with 31.2 per 100 000 in adult single males). While the lowest suicide rate observed among both age groups was in older widowed females: 3.5 per 100 000 (compared with 15.3 per 100 000 in adult widowed females). Across both age and sex groups, a lower suicide rate was observed among those who were married (14.1 per 100 000 in older males and 5.6 per 100 000 in older females).

Online supplemental file 5 provides Poisson regression analysis by marital status and age group. Between 2015 and 2020, older adults who were single (IRR: 2.44, 95% CI 2.02 to 2.94, p<0.05) or separated/divorced (IRR: 1.95, 95% CI 1.52 to 2.50, p<0.05) had higher suicide rates compared with married older adults. While older adults who were widowed had lower suicide rates compared with those who were married (IRR: 0.70, 95% CI 0.54 to 0.90, p<0.05). In contrast, among adults aged 18–59 years, single, separated/divorced and widowed individuals had higher suicide rates compared with married adults.

### Methods of suicide

There were age and sex differences in methods of suicide between adults aged 18 and 59 years and older adults (see table 1). Overall across males and females, drowning (OR: 2.34, 95% CI 1.86 to 2.96 p<0.001) and firearm-related suicides (OR: 2.07, 95% CI 1.40 to 3.07, p<0.001) account for a higher proportion of deaths in older adults compared with adults. Hanging was a less common method of death among older adults compared with adults (OR: 0.44, 95% CI 0.38 to 0.53, p<0.001). For males, the odds of drowning and firearm-related suicides were more than twice as high in those aged 60 years and over compared with adults. A similar pattern was observed among females: older females had almost three times the odds of drowning and nearly twice the odds of poisoning compared with younger females. In contrast, hanging was less common among older females than among those aged 18–59 years.

**Table 1:** Methods of suicide by age groups and sex

	Male						Female					
Method	60 and older		18–59 years		Older vs younger		60 and older		18–59 years		Older vs younger	
	N	%	N	%	OR	95% CI	N	%	N	%	OR	95% CI
Drowning	76	15.7	173	8.0	2.12*	1.59 to 2.84	51	30.2	94	13.1	2.87*	1.93 to 4.25
Hanging	264	54.4	1493	69.4	0.53*	0.43 to 0.64	44	26.0	413	57.8	0.26*	0.18 to 0.37
Firearm	38	7.8	80	3.7	2.20*	1.48 to 3.29	0	0	<10	<10	–	–
Poisoning	54	11.1	192	8.9	1.28	0.93 to 1.76	52	30.8	143	19.9	1.79*	1.23 to 2.60
Other	53	10.9	214	9.9	1.11	0.81 to 1.53	22	13.0	63	8.8	1.56	0.93 to 2.61

* p<0.001.

### Sociodemographic characteristics

Sociodemographic characteristics differed between older adults and those aged 18 and 59 years (table 2). The sex distribution was similar across age groups, with approximately three-quarters of deaths being male in both groups. In contrast, relationship status varied significantly: nearly half of older adults were married, cohabiting or in a civil partnership, compared with only one-third of younger adults, while younger adults were more likely to be single. The number of separated, divorced or widowed individuals was also higher among older adults. Living situation differed by age, with older adults more likely to live alone. Employment status differed by age group, with older adults more likely to be out of the labour force, whereas adults aged 18–59 years more frequently employed or unemployed. Socioeconomic group varied by age: individuals aged ≥60 years were more frequently farmers (14.1% vs 3.2%) and employers/managers (5.7% vs 3.7%) compared with those aged 18–59 years. In contrast, younger individuals were more often classified as non-manual workers (10.3% vs 7.2%) and as all other gainfully occupied or unknown (23.2% vs 18.2%). Other socioeconomic groups showed broadly similar distributions across age groups. All differences, except sex, were statistically significant.

**Table 2:** Characteristics of death by suicide by age groups

Characteristics		60 and older (n=654)		18–59 years (n=2868)		P value
		N	%	N	%	
**Sociodemographic**	**Sex** Male Female	485 169	74.2 25.8	2152 716	75.0 25.0	0.661
	**Relationship status** Married/cohabiting/civil part Single Separated/divorced Widowed	322 166 76 72	50.6 26.1 11.9 11.3	955 1579 214 32	34.4 56.8 7.7 1.2	<0.001
	**Living situation** Alone Family household* Other/unknown†	243 345 66	37.2 52.8 10.1	643 1668 557	22.4 58.2 19.4	<0.001
	**Employment status** In paid employment Unemployed Not in the labour market Unknown	144 59 376 75	22.0 9.0 57.5 11.5	1119 827 436 486	39.0 28.9 15.2 16.9	<0.001
	**Socioeconomic group** Agricultural workers All others gainfully occupied Employers and managers Farmers Higher professional Lower professional Manual skilled Non-manual Not recorded Own account worker Semi-skilled Unskilled	11 119 37 92 27 36 88 47 95 16 62 24	1.7 18.2 5.7 14.1 4.1 5.5 13.5 7.2 14.5 2.4 9.5 3.7	28 664 107 91 116 170 440 294 477 54 302 125	1.0 23.2 3.7 3.2 4.0 5.9 15.3 10.3 16.6 1.9 10.5 4.4	<0.001
Clinical	**History mental health** Yes No Unknown	434 30 190	66.4 4.6 29.1	1908 146 814	66.5 5.1 28.4	0.844
	**Mental health medication** Yes No Unknown	292 52 310	44.6 8.0 47.4	1069 308 1491	37.2 10.7 52.0	<0.001
	**Alcohol use history** Yes No	76 578	11.6 88.4	395 2473	13.8 86.2	0.146
	**Drug use history** Yes No	30 624	4.6 95.4	918 1950	32.0 68.0	<0.001
	**Prior self-harm history** Yes No Unknown	130 83 441	19.9 12.7 67.4	706 289 1873	24.6 10.1 65.3	0.012
	**Contact with health services** Yes No Unknown	434 11 209	66.4 1.7 32.0	1533 61 1274	53.4 2.1 44.5	<0.001
Death detail	**Coroner’s verdict** Beyond reasonable doubt Balance of probabilities	490 164	74.9 25.1	2130 738	74.3 25.7	0.708
	**Suicide note** Yes No Unknown	167 197 290	25.5 30.1 44.3	795 936 1137	27.7 32.6 39.6	0.088
	**Place of incident** Private Public Other	446 192 16	68.2 29.4 2.4	1875 922 62	65.6 32.2 2.2	0.348

* Family household includes living with partner/spouse, partner and children, children only and family.

† Other/unknown includes homeless/hostel, other shared, student accommodation and unknown.

‡ Missing data: Marital status n=106; place of incident n=9.

§ Unknown: Coroners report unknown when they have not been able to collect evidence to provide a definitive answer, and can neither confirm or deny presence of a characteristic.

### Clinical characteristics and death details

Clinical characteristics differed between older adults and those aged 18–59 years (table 2). The proportion with a history of mental health problems was similar across age groups, but older adults were more likely to be prescribed mental health medication, though this finding is based on 50% of the sample given that the remainder 50% was unknown. Alcohol use history did not differ significantly by age, whereas drug use history was lower among older adults. Prior self-harm was slightly less common in older adults, though this finding is based on less than 40% of the total study population as 60% was unknown. Contact with health services was higher in the older age group, although this finding is based on 60% of the total sample. In terms of death details, coroner’s verdict, presence of a suicide note and place of incident did not differ significantly between age groups.

### Adverse life events

Adverse life events at the time of death differed significantly by age group (table 3). Compared with those aged 18–59 years, older adults aged were more likely to have a documented physical health condition (35.9% vs 15.8%, p<0.001), recent acute pain (5.8% vs 1.3%, p<0.001), current mental health symptoms (39.1% vs 34.5%, p=0.03) and fears related to their own health (7.2% vs 1.2%, p<0.001). Loneliness or social isolation (3.4% vs 1.5%, p<0.001) and having seen a general practitioner (GP) in the month prior to death (3.4% vs 1.8%, p=0.01) were also more frequently recorded among older adults. In contrast, younger individuals more commonly had suicide exposure, financial concerns, work or study stress, interpersonal conflict, romantic relationship difficulties and overall relational difficulties (all p<0.05).

**Table 3:** Adverse life events of death by suicide by age groups

Adverse life events	60 and older (n=654)		18–59 years (n=2868)		P value
	N	%	N	%	
Physical health condition					
Mentioned Not mentioned	235 419	35.9 64.1	454 2414	15.8 84.2	<0.001
Recent acute pain					
Mentioned Not mentioned	38 616	5.8 94.2	37 2831	1.3 98.7	<0.001
Current mental health symptom					
Mentioned Not mentioned	256 398	39.1 60.9	990 1878	34.5 65.5	0.03
Fears about personal health					
Mentioned Not mentioned	47 607	7.2 92.8	34 2834	1.2 98.8	<0.001
Suicide exposure (including behaviour)					
Mentioned Not mentioned	10 644	1.5 98.5	86 2782	3.0 97.0	0.04
Financial concerns					
Mentioned Not mentioned	20 634	3.1 96.9	183 2685	6.4 93.6	<0.001
Work or study stress					
Mentioned Not mentioned	20 634	3.1 96.9	276 2592	9.6 90.4	<0.001
Interpersonal conflict					
Mentioned Not mentioned	36 618	5.5 94.5	424 2444	14.8 85.2	<0.001
Romantic relationship					
Mentioned Not mentioned	49 605	7.5 92.5	627 2241	21.9 78.1	<0.001
Relational difficulties (including romantic)					
Mentioned Not mentioned	64 590	9.8 90.2	833 2035	29.0 71.0	<0.001
Loneliness/isolation					
Mentioned Not mentioned	23 631	3.5 96.5	44 2824	1.5 98.5	<0.001
Seen GP in the last month					
Mentioned Not mentioned	22 632	3.4 96.6	51 2817	1.8 98.2	0.01

GP, general practitioner.

## Discussion

### Summary of findings

This national study examines suicide among older adults in Ireland over a 6-year period, compared with adults aged 18–59 years. Older adults had lower overall suicide rates (12.0 per 100 000 vs 17.5 per 100 000), although within-group variation existed. In particular, older single males had the highest suicide rate observed across all subgroups (38.1 per 100 000), highlighting substantial heterogeneity within the older population. Among older adults, older males aged 60–69 years had the highest age-specific suicide rate (21.8 per 100 000). Suicide rates among older adults remained relatively stable over the 6-year study period, with some variation by age and sex. Significant reductions were observed primarily among those aged 60–69 years, particularly among males in 2020 and females in 2016. Seasonal variation was observed, with suicide rates increasing during summer in both age groups; however, this increase was more pronounced among older adults than adults aged 18–59 years, particularly males. Age-related differences were observed across socioeconomic and clinical contexts. Older adults were more frequently represented in agricultural, farming and managerial groups, more likely to be married, live alone, have recent contact with GPs and less likely to have documented drug use. Adverse life events preceding death also differed by age: older adults were more likely to experience physical health conditions, acute pain, health-related fears, loneliness or social isolation, while adults more commonly experienced psychosocial stressors. Age and sex differences were observed in methods of suicide, with drowning and firearm-related suicides, in particular older males, accounting for a higher proportion of death compared with 18–59 year-olds. Taken together, these findings highlight the distinct profile of older adults who die by suicide in Ireland.

### Interpretation in the context of existing literature

Our findings are consistent with global evidence showing that suicide in later-life is characterised by different demographic and clinical features compared with younger adults.^16^ The finding of elevated suicide rates among older males aligns with long-standing research showing that older males are among the highest-risk demographic groups globally, often attributed to factors such as physical illness, functional decline, social isolation and lower rates of help-seeking, often shaped by cultural expectations and systemic barriers.^6 17^ Globally, suicide rates are reported to be highest among the 80 years and older age group,^2^ while our research indicated lower rates among the oldest age groups, particularly older females. The previous Irish study analysing suicide rates among older adults from 1997 to 2006 found similar suicide rates compared with our study (19.1 per 100 000 for males and 5.9 per 100 for females).^12^ Reduction in suicides observed in 2020 are consistent with previous international research which showed initial reductions during the first year of the COVID-19 pandemic,^18^ though further Irish research is needed to see if this reduction was sustained in further years after the pandemic.

Our findings are consistent with previous research^19 20^ demonstrating seasonal variation in suicide, with higher rates commonly reported during spring and summer months, particularly among males and older adults. Prior studies^21^ have similarly suggested that seasonal effects may be more pronounced in later-life, potentially reflecting the interaction between biological, environmental and social factors associated with ageing.

Farmers and agricultural workers have previously been identified as a group at risk of suicidal behaviour.^22 23^ Our study found that among the two age groups, older adults account for a larger proportion of farmers than 18–59 year-olds (14.1% vs 3.2%). This pattern may reflect the older age profile of the farming population in Ireland, where one in three farmers is aged 65 years and older.^24^ However, the over-representation of older adults within this occupational group requires particular attention, given the elevated suicide risk associated with farming, including factors such as social isolation and increased access to lethal means (eg, firearms), despite national legislation to reduce access to firearms.^25^

The predominance of drowning and firearm use among older adults mirrors patterns observed globally,^2^ suggesting that method choice may be influenced by generational, cultural and environmental factors, including access to firearms within rural communities.^6^ Globally, death by hanging is reported to be higher in older adults when compared with all ages.^2^ In contrast to this global trend, our study found lower deaths by hanging among older adults when compared with adults aged 18–59. For older females, the increased odds of poisoning align with evidence that older females often choose less violent methods.^26^

The sociodemographic profile identified in our study (higher rates of being widowed, living alone and being outside the labour market) reflects ageing trajectories but also overlaps with known risk factors for late-life suicide, including bereavement, social disconnection and role loss.^27^ A previous Irish study^28^ found that widowed people generally face higher suicide mortality but also noted an exception: widows aged 70 and over experienced lower suicide risk. Our study mirrors these findings, showing that older widowed females had the lowest suicide rates, despite the broader trend of increased risk among widowed individuals at younger ages. This increased risk was not observed among widowed adults aged 18–59 years. Consistent with previous literature,^29^ the higher proportion of older adults with health service contact, including seeing GPs in the last month, may highlight potential opportunities for detection and intervention in general practice and other services. Supporting this, analysis of the same data source^30^ found that 97% of individuals who died by suicide, across all ages, had contact with a healthcare professional in the year prior to death. In our study, older adults were more likely than younger adults to have recent GP contact, highlighting primary care as a key setting for the delivery of universal, selective and indicated interventions for older adults.

### Clinical implications

This study underscores the need for age-specific suicide prevention strategies. First, the high suicide rates among males aged 60–69 years highlight this group as a priority target for selective interventions. This age group is marked by transition from working life to retirement, which, while welcome to some, can be a significant stressor for others and contribute to mental health decline. Integrated approaches involving primary care, specialist care and social support services including social prescribing may be particularly important for identifying and delivering individual-level interventions to address clinical needs and lower risk in this group.

Second, the method-specific differences observed emphasise the importance of means restriction strategies tailored to older adults. This includes safe storage of firearms in rural households, enhanced surveillance in coastal and water-adjacent regions, and environmental modifications in community, residential and long-term care settings. As nearly one-third of older females died by poisoning, and older adults were more likely to be prescribed mental health medication, there may be a role for considering shorter prescriptions and other methods for stewardship of potentially lethal medications.

Third, the finding that older adults are more likely to have recent contact with health services, yet less likely to have known risk behaviours such as drug use history or prior self-harm, supports calls for improved routine screening for suicidality and psychosocial distress in older populations, particularly in healthcare settings. Clinicians, including frontline GPs, should be aware that older adults presenting with new functional decline, pain or social isolation may be at elevated suicide risk even in the absence of traditional warning signs and assess for self-harm and suicidal thoughts. These symptoms should not be overlooked, dismissed or attributed to normal ageing, and care needs to be taken in balancing the need for medications with making available potentially lethal means.

Finally, while the high proportion of older single males and older adults living alone highlights the importance of outreach and social prescribing initiatives, these efforts must be supported by adequate structural supports (eg, accessible transport) to ensure older adults can actually participate.

These findings have relevance for global suicide prevention efforts, including Sustainable Development Goal 3, which aims to reduce premature mortality from non-communicable causes, including suicide. By identifying age-specific and sex-specific patterns in suicide rates and circumstances in later-life, this study provides evidence to support targeted prevention strategies, improved detection within health services and monitoring of progress towards suicide reduction targets. Strengthening national surveillance of suicide in older populations is essential for informing policy and evaluating progress within global mental health frameworks.

### Strengths and limitations

Key strengths of this study include the use of a national suicide mortality dataset and case series design with a standardised application of criteria to determine cases reducing bias. The large sample size over a 6-year period, and the ability to compare older and younger adults across a wide range of sociodemographic and clinical characteristics were also strengths of this study. While a Lived Experience group contributed to the interpretation of findings, people with lived experience were not involved in shaping the research questions or selection of variables as this study involved secondary data analysis.

Several limitations should be acknowledged. First, although Ireland has a structured legal system for recording suicide verdicts, this study relies on coronial data, and classification practices may vary due to differences in thresholds, assessment processes and information available to coroners, which could differ between older and younger adults. To address potential underreporting, the IPSDS applies a research-determined suicide classification alongside the coroner-determined verdict, reducing the likelihood of so-called ‘hidden suicides’. Second, some variables had substantial levels of missing or unknown data, particularly in clinical history fields, including mental health and service-related, which may limit interpretability. For instance, information on adverse life events was subject to variability in documentation across coronial cases and may underestimate the presence or timing of such factors where they were not identified or recorded during the investigation. Third, although national-level data allow robust descriptive comparisons, causality cannot be inferred. Fourth, the small numbers among older females and the ‘oldest-old’ (≥80 years) limited statistical power for subgroup analyses, reducing our ability to detect significant trends over time. Due to the small population size and the rarity of suicide among older adults, it was not possible to examine further associations with specific clinical outcomes. Furthermore, the IPSDS only has 6 years of available data (2015–2020), which may not be sufficient time to fully detect changes in trends. Fifth, although the IPSDS is a rich dataset, key details such as ethnicity, deprivation levels and migrant status were not captured. This not only limits the ability to examine potential structural and social inequalities but also means that the experiences of some minoritised and migrant communities remain invisible within these findings. Finally, while coronial data are essential for understanding the circumstances of death, they provide only a partial account of a person’s life. These records cannot capture the full complexity of an individual’s relationships, identity or lived experience. We recognise that the data analysed represent real people and approach this work with respect and care.

## Conclusions and future directions

Suicide among older adults in Ireland presents a distinct profile compared with adults, characterised by different sociodemographic backgrounds, clinical characteristics and methods of suicide. Older adults aged 60–69 years, especially single males and those transitioning to retirement, represent a key group for targeted prevention. Strengthening detection in healthcare settings, including frontline general practice, addressing social isolation and implementing age-tailored means restriction strategies are essential components of a comprehensive suicide prevention approach for older adults.

Future research should focus on better understanding pathways to suicide in later-life, including the roles of physical illness, loneliness and transitions such as retirement. Improved clinical documentation and linkage of health and social care data may also enhance risk detection and prevention efforts. As Ireland’s population continues to age, developing a coordinated, evidence-based and person-centred framework for late-life suicide prevention is increasingly important.

## Supplementary material

10.1136/bmjment-2026-302665 online supplemental file 1
