# Supplementary material for: Suicide among older adults in Ireland: a national case series of coronial data, 2015–2020
Source: BMJ Ment Health. 2026 Jul 14;29(1):e302665. doi: 10.1136/bmjment-2026-302665 (PMC13374407; doi:10.1136/bmjment-2026-302665)
Supplement: online supplemental file 1 [file bmjment-29-1-s001.docx]

Supplementary File 1. Suicide rate among adults aged 18-59 years 2015-2020

Supplementary File 2. Poisson regression analysis of annual change in rate of suicide in older adults

1. Older adults aged 60 years and older: 2015-2020

| Year | Male IRR (95% CIs) | Female  IRR (95% CIs) | All  IRR (95% CIs) |
| --- | --- | --- | --- |
| 2015 | 1.00 (Ref) | 1.00 (Ref) | 1.00 (Ref) |
| 2016 | 0.92 (0.68-1.25) | 0.62 (0.35-1.08) | 0.84 (0.64-1.10) |
| 2017 | 0.81 (0.59-1.10) | 0.73 (0.43-1.24) | 0.79 (0.66-1.03) |
| 2018 | 0.97 (0.72-1.31) | 1.12 (0.70-1.80) | 1.02 (0.79-1.31) |
| 2019  2020 | 0.89 (0.66-1.20)  0.73 (0.53-0.99)* | 0.69 (0.40-1.17)  0.87 (0.53-1.43) | 0.84 (0.65-1.09)  0.77 (0.59-1.01) |

*p<0.05

1. Older adults aged 60-69 years: 2015-2020

| Year | Male IRR (95% CIs) | Female  IRR (95% CIs) | All  IRR (95% CIs) |
| --- | --- | --- | --- |
| 2015 | 1.00 (Ref) | 1.00 (Ref) | 1.00 (Ref) |
| 2016 | 0.94 (0.64-1.37) | 0.46 (0.22-0.94)* | 0.80 (0.57-1.11) |
| 2017 | 0.81 (0.55-1.20) | 0.79 (0.43-1.45) | 0.80 (0.58-1.12) |
| 2018 | 1.04 (0.72-1.50) | 0.93 (0.52-1.67) | 1.01 (0.74-1.38) |
| 2019  2020 | 0.80 (0.54-1.18)  0.64 (0.42-0.96)* | 0.88 (0.49-1.57)  0.80 (0.44-1.44) | 0.82 (0.59-1.13)  0.69 (0.49-0.96)* |

*p<0.05

1. Older adults aged 70-79 years: 2015-2020

| Year | Male IRR (95% CIs) | Female  IRR (95% CIs) | All  IRR (95% CIs) |
| --- | --- | --- | --- |
| 2015 | 1.00 (Ref) | 1.00 (Ref) | 1.00 (Ref) |
| 2016 | 0.89 (0.50-1.60) | 0.94 (0.33-2.67) | 0.90 (0.54-1.50) |
| 2017 | 0.74 (0.41-1.37) | 0.65 (0.21-2.06) | 0.73 (0.42-1.24) |
| 2018 | 0.97 (0.55-1.70) | 1.37 (0.53-3.5) | 1.07 (0.66-1.73) |
| 2019  2020 | 1.11 (0.65-1.90)  0.95 (0.55-1.65) | 0.24 (0.05-1.16)  1.12 (0.43-2.95) | 0.91 (0.56-1.50)  1.00 (0.62-1.61) |

*p<0.05

1. Older adults aged 80 years and older: 2015-2020

| Year | Male IRR (95% CIs) | Female  IRR (95% CIs) | All  IRR (95% CIs) |
| --- | --- | --- | --- |
| 2015 | 1.00 (Ref) | 1.00 (Ref) | 1.00 (Ref) |
| 2016 | 0.94 (0.30-2.91) | 1.93 (0.17-21.3) | 1.09 (0.39-3.01) |
| 2017 | 1.06 (0.36-3.14) | 1.68e-08 (0-.) | 0.93 (0.33-2.65) |
| 2018 | 0.57 (0.16-2.03) | 3.70 (0.41-33.01) | 1.03 (0.37-2.83) |
| 2019  2020 | 0.95 (0.32-2.84)  0.65 (0.20-2.14) | 1.58e-08 (0-.)  0.89 (0.10-14.20) | 0.86 (0.30-2.46)   - 1. (0.24-2.15) |

*p<0.05

1. Adults aged 18-59 years: 2015-2020

| Year | Male IRR (95% CIs) | Female  IRR (95% CIs) | All  IRR (95% CIs) |
| --- | --- | --- | --- |
| 2015 | 1.00 (Ref) | 1.00 (Ref) | 1.00 (Ref) |
| 2016 | 1.02 (0.89-1.18) | 0.91 (0.69-1.18) | 1.01 (0.89-1.15) |
| 2017 | 0.95 (0.83-1.10) | 0.94 (0.72-1.22) | 0.95 (0.84-1.08) |
| 2018 | 0.96 (0.83-1.11) | 1.20 (0.94-1.54) | 1.02 (0.90-1.15) |
| 2019  2020 | 0.90 (0.78-1.04)  0.81 (0.70-0.94)* | 1.02 (0.79-1.32)  1.11 (0.86-1.43) | 0.95 (0.84-1.08)  0.88 (0.77-1.00) |

*p<0.05

Supplementary File 3. Poisson regression analysis by season

1. Older adults aged 60 and older: 2015-2020

| Season | Male IRR (95% CIs) | Female  IRR (95% CIs) | All  IRR (95% CIs) |
| --- | --- | --- | --- |
| Winter | 1.00 (Ref) | 1.00 (Ref) | 1.00 (Ref) |
| Spring | 1.18 (0.90-1.57) | 1.32 (0.80-2.18) | 1.23 (0.97-1.57) |
| Summer | 1.34 (1.06-1.69)* | 1.49 (0.93-2.38) | 1.38 (1.13-1.68)* |
| Autumn | 1.17 (0.92-1.49) | 0.94 (0.53-1.65) | 1.11 (0.90-1.36) |

*p<0.05

1. Adults aged 18-59 years: 2015-2020

| Season | Male IRR (95% CIs) | Female  IRR (95% CIs) | All  IRR (95% CIs) |
| --- | --- | --- | --- |
| Winter | 1.00 (Ref) | 1.00 (Ref) | 1.00 (Ref) |
| Spring | 1.09 (0.98-1.20) | 1.17 (0.96-1.42) | 1.09 (0.98-1.20) |
| Summer | 1.11 (1.01-1.23)* | 1.22 (0.98-1.50) | 1.11 (1.01-1.23)* |
| Autumn | 1.10 (0.99-1.21) | 1.18 (0.97-1.46) | 1.10 (0.99-1.21) |

*p<0.05

Supplementary File 4. Suicide rate by marital status and age group, 2015-2020

*Married includes co-habiting and civil partnership; Separated includes divorced

Supplementary File 5. Poisson regression analysis by marital status

a. Older adults aged 60 and older: 2015-2020

| Marital status | Male IRR (95% CIs) | Female  IRR (95% CIs) | All  IRR (95% CIs) |
| --- | --- | --- | --- |
| Married | 1.00 (Ref) | 1.00 (Ref) | 1.00 (Ref) |
| Single | 2.71 (2.20-3.33)* | 1.58 (1.02-2.43)* | 2.44 (2.02-2.94)* |
| Separated or divorced | 1.92 (1.41-2.61)* | 2.32 (1.50-3.62)* | 1.95 (1.52-2.50)* |
| Widowed | 1.33 (0.97-1.83) | 0.62 (0.40-0.96)* | 0.70 (0.54-0.90)* |

*p<0.05

b. Adults aged 18-59 years: 2015-2020

| Marital status | Male IRR (95% CIs) | Female  IRR (95% CIs) | All  IRR (95% CIs) |
| --- | --- | --- | --- |
| Married | 1.00 (Ref) | 1.00 (Ref) | 1.00 (Ref) |
| Single | 1.59 (1.45-1.75)* | 1.71 (1.45-2.01)* | 1.67 (1.54-1.81)* |
| Separated or divorced | 1.87 (1.56-2.23)* | 2.17 (1.67-2.82)* | 1.79 (1.54-2.08)* |
| Widowed | 1.69 (1.01-2.82)* | 2.56 (1.57-4.19)* | 1.63 (1.14-2.31)* |

*p<0.05
